# Exploring the Relationship Between Early Postnatal Weight Gain and the Severity of Retinopathy of Prematurity: Insights From a Tertiary Care Facility in Central India

**DOI:** 10.7759/cureus.81262

**Published:** 2025-03-26

**Authors:** Bhanu Priya, Vijaya Sahu, Phalguni Padhi, Aseem Kumar

**Affiliations:** 1 Ophthalmology, Centre for Sight, New Delhi, IND; 2 Ophthalmology, All India Institute of Medical Sciences, Raipur, Raipur, IND; 3 Neonatology, All India Institute of Medical Sciences, Raipur, Raipur, IND

**Keywords:** low birth weight, number of days oxygen administered, postnatal weight gain, prematurity, retinopathy of prematurity, screening

## Abstract

Purpose

The primary aim of this study is to ascertain whether early postnatal weight gain serves as an independent predictor for the severity of retinopathy of prematurity (ROP) in preterm infants.

Method

In this prospective observational study, 50 preterm infants (<34 weeks GA at birth or <2000 gm BW) in the Neonatal Care Unit were screened for ROP from June 2021 to June 2022. All infants were assessed weekly for postnatal weight gain during the first six weeks of life and the results were correlated with the severity of ROP.

Result

Out of 50 preterm infants, 25 (50%) developed ROP, and 25 (50%) had no ROP. Type 1 ROP was identified in two (8%) preterm infants. The mean gestational age and birth weight for the ROP group were 29.98 ± 1.80 weeks (SEM 95% CI) and 1161.32 ± 340.55 gm (SEM 95% CI), which were lower than those of the No ROP group, which had 31.94 ± 1.71 weeks (SEM 95% CI) and 1359.80 ± 313.52 gm (SEM 95% CI), respectively. The change in weight (grams) between the ROP and No ROP groups was statistically significant each week, except for the first week. However, for the first six weeks, no statistical correlation was found between Type 1 ROP and Type 2 ROP regarding weight change.

Conclusion

This study posits that postnatal weight gain, particularly evident in the fourth week, may be a pivotal factor in predicting the onset and severity of ROP in preterm infants. Further research and analysis are required to validate these findings and enhance our understanding of the relationship between postnatal growth patterns and ROP development.

## Introduction

Retinopathy of prematurity (ROP) is a vaso-proliferative disorder of the retina among preterm babies, first described in 1942 and continues to be the most important avoidable cause of childhood blindness in both developed and developing countries. It is a severe consequence of prematurity and can lead to blindness unless recognized and treated early [1]. Approximately 15 million babies are born preterm worldwide each year, and India has the highest number of preterm births [1]. Out of 26 million annual live births in India, approximately 8.7% of newborns weigh less than 2000 grams. This would imply that almost two million newborns are at risk for developing ROP [2]. Studies from India have reported ROP in 20% to 52% of screened neonates [3]. More recent studies report lower rates of ROP, ranging from 20% to 30% [3]. In a global estimate of ROP in 2010, a total of 184,700 preterm babies developed any form of ROP; of this, 20,000 became blind, and a further 12,300 were visually impaired from ROP [4]. India attributed nearly 10% of global estimates of ROP-related visual impairment in 2010, and a higher incidence of ROP is reported in India (19.3%-47.2%) compared to Western countries (1.2%-19.8%) [1,4].

Numerous interconnected risk elements have a correlation with the intensity of ROP. Risk factors are categorized as non-modifiable (low birth weight, prematurity, number of days oxygen administered) and modifiable (blood transfusions, respiratory distress syndrome (RDS), sepsis, intraventricular hemorrhage (IVH), intrauterine growth retardation, vitamin E deficiency, anemia, seizures, postnatal weight gain) [1].

As ROP is a dynamic, time-bound disease that is not present at birth, we could focus on non-modifiable factors to increase the predictability of disease occurrence and timely intervention. Various affordable and inventive approaches have been formulated for risk categorization, utilizing sequential weight gain measurements after birth. Weight increase is a recognized proxy for the presence of insulin-like growth factor 1 (IGF-1), with continuous diminished neonatal serum IGF-1 levels in premature infants correlating with inadequate weight growth. Additionally, decreased serum IGF-1 levels lead to insufficient retinal vascular endothelial growth factor (VEGF) stimulation. This leads to inadequate development of the blood vessels in the retina during the early stages of life after birth, resulting in the onset of ROP. Sethi et al. [5], in their analysis, studied the relationship between weight gain in infants and its relationship with the occurrence of ROP. In their analysis of 62 ROP babies, they concluded that poor weight gain of less than 29.33 g/day was a high risk for ROP, and babies with weight gain of 21.91 g/day were at high risk for severe ROP. The babies should be followed meticulously, and the rate of weight gain in these preterm babies will help prioritize them. Wongnophirun et al. [6] investigated the association between relative weight gain and severity of ROP in very low birth weight infants and found that poor weight gain and low-calorie intake during the second week of age were associated with severe ROP. The children with very low birth weight required laser treatment. Weight gain and calorie intake during this period are essential factors and may improve the outcome of ROP. According to the best literature review, few previous authors have studied the relationship of low birth weight with the development of ROP, but none have studied its relationship with the severity of ROP. Taking this as our research question, we wanted to explore the relationship between early postnatal weight gain and the severity of ROP from central India. We hypothesize that slower early postnatal weight gain is significantly associated with the severity of ROP. To the best of our knowledge, this is the first study documenting the relationship between early postnatal weight gain and the severity of ROP from central India.

## Materials and methods

This prospective observational study was conducted in the Department of Ophthalmology and Neonatal Intensive Care Unit (NICU) at a tertiary care facility from June 2021 to June 2022. Study approval was obtained from the Institutional Review Board of the AIIMS, Raipur, Institutional Ethics Committee (no. 1729/IEC-AIIMSRPR/2021). Written informed consent was obtained from the parents of preterm infants, ensuring confidentiality. The study was conducted in compliance with Good Clinical Practices and consistent with the tenets of the Declaration of Helsinki, including International Harmonization Guidelines. All preterm infants with a gestational age <34 weeks and/or a birth weight <2000 gm, admitted to the NICU, were screened for ROP, both inborn and outborn (as per the Rashtriya Bal Swasthya Karyakaram, or RBSK). RBSK is a program under the national health mission in India to screen and manage children up to the age of 18 years for birth defects, diseases, deficiencies, and developmental delays. Preterm infants with congenital anomalies, suspected inborn errors of metabolism, or those who died between the first and sixth ophthalmologic examinations were excluded from the study. Detailed history regarding demographic and socioeconomic information, baseline neonatal characteristics, and perinatal and postnatal history was documented. All preterm infants underwent ROP screening by a senior ophthalmologist (VKS) with a minimum of 10 years of experience. The screening was done under topical anesthesia using proparacaine eye drops. The first screening for ROP was done within the fourth postnatal week, or the second or third postnatal week for very small preterm infants with a gestational age <28 weeks or <1200 gm. Fundus evaluation was done by a single ophthalmologist using an indirect ophthalmoscope after dilatation of pupils with tropicamide 0.5% and phenylephrine 2.5%, one drop at 15-minute intervals, three times, with fasting for one hour after the last drop. The staging of ROP was done according to the International Classification of ROP (ICROP) [7].

Weight measurements were done without the clothing of preterm babies by an electronic weighing machine in the NICU. Outborn preterm infants, whose initial management was done outside, had their weight taken from the available documents. The infant's medical records carefully documented all the postnatal events, such as apnea, sepsis, necrotizing enterocolitis (NEC), and postnatal management, i.e., oxygen supplementation, continuous positive airway pressure (CPAP) care, mechanical ventilation, and blood transfusion. Follow-up was done weekly until six weeks of postnatal age for postnatal weight measurement and fundus evaluation, and thereafter, according to the severity of ROP as per ICROP guidelines. Babies were categorized into Type 1 ROP and Type 2 ROP [7]. Type 1 ROP is defined as any ROP with plus disease in Zone I, Stage 3 ROP in Zone I, and Stage 2 or Stage 3 ROP with plus disease in Zone II. Type 2 ROP is a milder form of ROP (Stage 1 or 2 in Zone II or III), with or without pre-plus disease, and it does not require treatment [8].

All infants were managed according to the severity of ROP, with termination of screening at 45 weeks of postmenstrual age or upon attainment of a mature retina, or until complete remission of ROP after treatment. The primary outcome measure was to evaluate whether poor postnatal weight gain is an independent risk factor for predicting the severity of ROP in preterm infants. The secondary outcome measures were a comparison of the severity of ROP between inborn and outborn preterm babies admitted to the NICU at our tertiary care facility, and the association of the severity of ROP in preterm infants with other risk factors, i.e., sepsis, duration of oxygen exposure (ventilation, CPAP, hood), blood transfusion, RDS, and IVH.

Statistical analysis

Statistical analysis was carried out using SPSS for Windows, Version 16.0 (Released 2007; SPSS Inc., Chicago, IL, USA). Continuous and categorical variables were expressed as mean ± SD and percentages. The Chi-square test was used to compare categorical variables. Sensitivity, specificity, positive predictive value (PPV), and negative predictive value (NPV) were calculated, and the receiver operating characteristic (ROC) curve was generated to determine the cut-off value. Two-sided p-values were considered statistically significant at p < 0.05.

## Results

During the study period, 62 inborn and five outborn preterm infants were assessed, but after exclusion, 48 inborn and two outborn preterm infants (a total of 50) were included and followed up. Out of 50 preterm babies, 22 (44.0%) were males and 28 (56.0%) were females. The male:female ratio was 0.8:1. In the present study, the mean gestational age (±SD) and birth weight (±SD) were 30.96 ± 2.00 weeks and 1260.56 ± 339.11 gm, respectively. Out of 50 preterm babies, nine (18%) were extremely low birth weight (<1000 gm), and four (8%) were extremely preterm (<28 weeks). A total of 25 (50%) babies had ROP; out of that, 23 (92%) were type 2 ROP, and the incidence of development of type 1 ROP requiring treatment was two (8%). Among the 25 preterm babies who developed ROP, 13 (54.2%) developed Stage 1 ROP, 10 (41.7%) developed Stage 2 ROP, one (4.2%) developed Stage 3 ROP, and only one (4%) developed aggressive posterior-retinopathy of prematurity (AP-ROP). Type 1 ROP was seen in two (8%) preterm babies: one was inborn and developed Zone 2 Stage 3 for 5 clock hours with plus disease, and laser photocoagulation was done. Another preterm baby developed Type 1 ROP (AP-ROP), for which an intravitreal anti-VEGF injection of ranibizumab (0.25 mg/0.025 mL) was given.

We also analyzed various risk factors for the development of ROP (Table 1), and a univariate regression analysis of these risk factors was done (Table 2).

**Table 1:** Association between ROP and risk factors (n = 50) CPAP: continuous positive airway pressure; MV: mean volume

Risk Factor	ROP		p-value
	Yes (n = 25)	No (n = 25)	
Prematurity	25 (100.0%)	25 (100.0%)	1
Perinatal Asphyxia	6 (24.0%)	3 (12.0%)	0.463
Mode of Oxygen Delivery			0.009
Room Air	2 (8.0%)	6 (24.0%)	
Nasal Prong/Hood	0 (0.0%)	3 (12.0%)	
CPAP	17 (68.0%)	16 (64.0%)	
MV	6 (24.0%)	0 (0.0%)	
Duration of Oxygenation			0.002
<24 Hours	6 (27.3%)	0 (0.0%)	
1-5 Days	2 (9.1%)	11 (57.9%)	
5-10 Days	11 (50.0%)	6 (31.6%)	
>10 Days	3 (13.6%)	2 (10.5%)	
Sepsis	6 (24.0%)	4 (16.0%)	0.48
Intraventricular Hemorrhage	2 (8.0%)	3 (12.0%)	1
Blood Transfusion	3 (12.0%)	2 (8.3%)	1
Respiratory Distress Syndrome	23 (92.0%)	19 (76.0%)	0.247

**Table 2:** Risk factors for the development of ROP, univariate analysis (n = 50) ROP: retinopathy of prematurity

S. No.	Risk Factor	ROP (n = 25)	No ROP (n = 25)	Significance (p-value)
1	Female, %	44	68	0.087
2	Small for Gestational Age (SGA), %	20	32	0.345
3	Mean Gestational Age (Weeks) (Mean ± SEM, 95% CI)	29.98 ± 1.80	31.94 ± 1.71	0.001
4	Mean Birth Weight (Gram) (Mean ± SEM, 95% CI)	1161.32 ± 340.55	1359.80 ± 313.52	0.033
5	Mode of Oxygen Delivery, %	NA	NA	0.012
	Nasal Prong/Hood	0.0	12	
	Continuous Positive Airway Pressure (CPAP)	68	64	
	Mean Volume (MV)	24	0	
6	Duration of Oxygenation, %	NA	NA	0.002
	<24 Hours	27.3	0.0	
	1-5 Days	9.1	57.9	
	5-10 Days	50	31.6	
	>10 Days	13.6	10.5	
7	Asphyxia, %	24	12	0.463
8	Sepsis, %	24	16	0.480
9	Intraventricular Hemorrhage (IVH), %	8	12	1.000
10	Blood Transfusion, %	12	8.3	1.000
11	Respiratory Distress Syndrome (RDS), %	92	76	0.247

On univariate regression analysis of change in weight (gm) with the ROP group at each week for the first six weeks of life, it was calculated. It showed that weight change (gm) was significantly correlated with the development of ROP each week, especially in the fourth week (Table 3).

**Table 3:** Univariate regression analysis of change in weight (gm) with ROP group ROP: retinopathy of prematurity

S. No.	Time Point (Weeks)	Cut-Off (gm)	Odd's Ratio	95% CI	p-value
1	1st	< -100	5.09	1.45-17.92	0.009
2	2nd	< 20	3.16	1.00-10.03	0.048
3	3rd	< 180	4.57	1.38-15.11	0.011
4	4th	< 410	12.46	2.41-64.49	< 0.001
5	5th	< 455	5.46	1.63-18.36	0.005
6	6th	< 520	7.11	1.99-25.47	0.002

**Figure 1:**
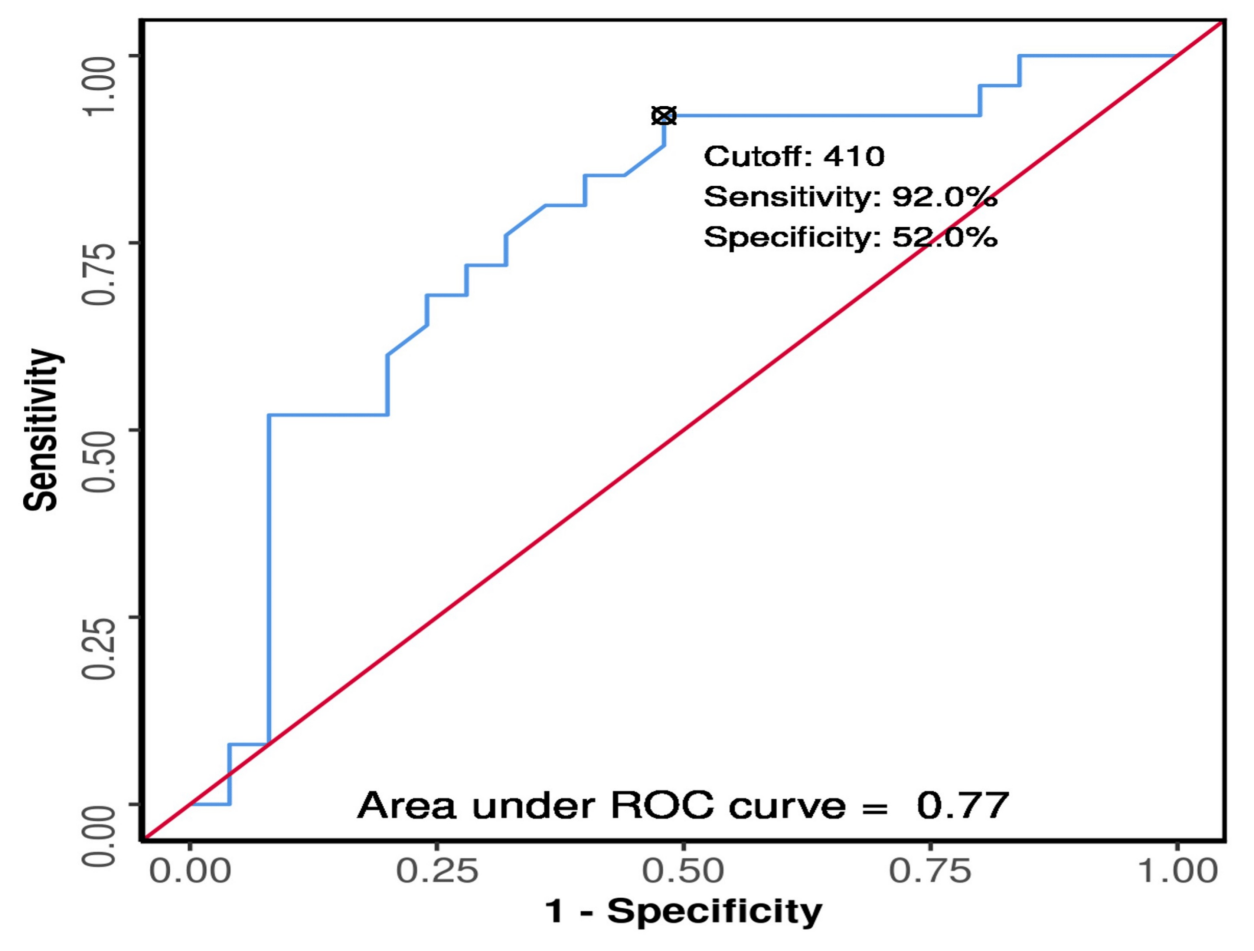
ROC curve for mean change in weight and development of ROP in four weeks ROC: receiver operating characteristic; ROP: retinopathy of prematurity

The ROC curve showed the discriminative cut-off value of 410 gm in the fourth week (odds ratio: 12.46; 95% CI: 2.41-64.49, p < 0.001), with a sensitivity of 92% and specificity of 52% for the development of ROP (Figure 1). In type 1 ROP cases, the minimum change was observed during the second week (-45.00 ± 77.78), and the maximum was observed during the sixth week (245.00 ± 77.78). For type 2 ROP cases, the minimum change was observed during the first week (-87.26 ± 64.39), and the maximum was observed during the sixth week (542.22 ± 263.25).

The babies who developed Type 1 ROP showed low weight gain compared to the Type 2 ROP group, but it was not statistically significant between the Type 1 and Type 2 ROP groups due to unequal sample distribution (Table 4).

**Table 4:** Comparison of change in weight (gm) over six weeks between Type 1 ROP and Type 2 ROP group ROP: retinopathy of prematurity

S. No.	Time Point (Week)	Change in Weight (Gram), (Mean ± SEM, 95% CI)		Significance (p-value)
		Type 1 ROP	Type 2 ROP	
1	1st	-150.00 ± 49.50	-87.26 ± 64.39	0.132
2	2nd	-45.00 ± 77.78	-7.52 ± 91.10	0.394
3	3rd	45.00 ± 134.35	104.22 ± 129.43	0.394
4	4th	110.00 ± 169.71	230.30 ± 156.99	0.367
5	5th	150.00 ± 141.42	372.17 ± 195.52	0.146
6	6th	245.00 ± 77.78	542.22 ± 263.25	0.088

## Discussion

ROP is one of the major causes of preventable blindness in both developed and developing countries [9]. Identifying postnatal factors that can predict who will develop severe ROP requiring treatment would be beneficial [10]. In this study, the primary focus was on postnatal weight gain during the first six weeks, correlating it with the severity of ROP. A total of 50 preterm babies (100%) were screened, of which 28 (56%) were female, and two (4%) were outborn. The overall percentage of ROP development was 50%. The findings of the current study were similar, though toward the upper range, to studies done across India, which documented an incidence between 20% and 51.9% [11-15]. The incidence of Type 1 ROP requiring treatment was two (8%) neonates, which is also comparable to prior studies [11,13,16-19].

A higher prevalence of severe ROP was reported by Charan et al. and Rekha and Battu, but these studies were done more than two decades ago, and there has been an improvement in neonatal practices since then [11,13]. This should have led to a decreased incidence of ROP, but this does not seem to be the case, as even recent reports show highly variable rates of severe ROP in different centers. Kumar et al. have reported a 4.7% incidence of severe ROP, and Vinekar et al. documented severe ROP requiring treatment in 3.5% [16,17]. On the other hand, Hungi et al. and Ahuja et al. have reported a higher incidence of 10.2% and 13.2% of severe ROP, respectively [18,19]. This is an indication that neonatal care is highly variable in different parts of our country, and there is a great divide between developed and less-developed parts.

Among the 25 preterm babies who developed ROP, 13 (54.2%) developed Stage 1 ROP, 10 (41.7%) developed Stage 2 ROP, one (4.2%) developed Stage 3 ROP, and only one (4.2%) developed AP-ROP. In one of the previous studies conducted by Akther et al., 33 neonates developed ROP, in which Stage 1 ROP was seen in 17 neonates (52%), Stage 2 ROP in six neonates (18%), Stage 3 ROP in one neonate (13%), and AP-ROP in eight neonates (24%), which is very comparable to our study [20]. However, our study had a much smaller number of AP-ROP cases. In another study by Rao et al., 21 neonates had Stage 1 ROP, 24 neonates had Stage 2 ROP, and 16 neonates had Stage 3 ROP, which is somewhat higher than our study, but they included 61 preterm babies [21].

In the present study, the mean gestational age (±SD) and birth weight (±SD) for all 50 included preterm babies were 30.96 ± 2.00 weeks and 1260.56 ± 339.11 gm, respectively. Out of 50 preterm babies, nine (18%) were extremely low birth weight (<1000 gm), and four (8%) were extremely preterm (<28 weeks). We found similar results in the study by Aydemir et al., with a mean gestational age of 29.3 ± 2.3 weeks and a mean birth weight of 1165 ± 223 gm [10]. In another study by Akther et al., the mean gestational age (±SD) was found to be 32.49 ± 1.6 weeks and the mean birth weight (±SD) to be 1446.44 ± 253.1 gm, which is somewhat higher than our study [20]. 

In the present study, the mean gestational age and birth weight for the ROP group were found to be 29.98 ± 1.80 (SEM 95% CI) weeks and 1161.32 ± 340.55 (SEM 95% CI) gm, which is similar to the study by Aydemir et al., but Wallace et al. found a lower mean gestational age and birth weight for the development of ROP than our study [10,22].

In the present study, the mean gestational age and birth weight for Type 1 ROP requiring treatment were similar to the study by Dwivedi et al., with a mean gestational age and birth weight for severe ROP of 31.05 ± 0.28 (SEM 95% CI) weeks and 1.34 ± 0.04 (SEM 95% CI) kg, respectively [15]. A lower mean gestational age and birth weight were recorded by Aydemir et al. (27.2 ± 1.9 weeks and 959 ± 185 g), Wallace et al. (26.1 ± 1.8 weeks and 827 ± 207 gm), and Kim et al. (26-29 weeks and 910 (770-1200) gm) [10,22,23]. In the present study, 92% of preterm babies with Type 2 ROP regressed spontaneously, and 8% of preterm babies with Type 1 ROP who required treatment, which is almost the same as Aydemir et al., who found 6%. However, Kumar et al. and Vinekar et al. documented Type 1 ROP in 4.7% and 3.5%, respectively, which is lower than our study [10,16,17]. Some studies, like Hungi et al. [18] and Ahuja et al. [19], reported a higher incidence of severe ROP, with 10.2% and 13.2%, respectively, which is higher than our study. This may be due to variable neonatal care in different parts of our country and different sample sizes in each study. In the present study, Type 1 ROP between inborn and outborn preterm babies was 1:1, but it was not significant. One outborn preterm baby was born outside, and the initial four days of management were done in another NICU. The first hour of postnatal management is critical and considered a golden period for developing ROP, as suggested by another study [24]. Yet, the small sample size does not permit us to draw conclusions on the development of ROP between inborn and outborn babies. It was also found that all nine (18%) extremely low birth weight preterm babies were inborn. Initial management was done in our institute’s NICU, and none of them developed severe ROP (Type 1). This may reflect good neonatal care in our institute. In the study by Pan et al. in 2021, no statistical difference in the presence of severe ROP (%) was found between inborn and outborn babies (1.9:1.5), comparable to our study [25].

In the present study, we also studied various risk factors for the development of ROP; univariate analysis of various risk factors - gestational age (p = 0.001), low birth weight (p = 0.018), and duration of oxygenation (p = 0.002) - had been found statistically significant, with a p-value <0.05. Gestational age, low birth weight, and oxygen delivery duration are well-established risk factors [3]. The mode of oxygen delivery has not been extensively studied. There are some studies that highlighted the use of various modes of oxygen delivery. In 2019, a study by Pastro and Toso [9] showed that not only duration but also the mode of oxygen delivery significantly affects the development of ROP. Our study also found a significant correlation between the mode of oxygen delivery and the development of ROP. In another study by Rao et al. [21], there was no significant correlation between the mode of oxygen delivery and the development of ROP. In the present study, all mechanically ventilated preterm babies developed ROP. In our institute, blenders were used for all preterm babies for oxygen supplementation. For many years, the high level of oxygen administered to neonates was considered the leading risk factor for ROP development [17]. However, the disease kept occurring even after careful control of oxygen administration [16]. In our institution, pulse oximetry always monitors oxygen administration, with recommended saturation between 88% and 92%. However, other risk factors, like sepsis (p = 0.480), IVH (p = 1.000), blood transfusion (p = 1.000), and RDS (p = 0.247), had not been found statistically significant in the present study, but Rao et al. [21] had found IVH and sepsis as significant risk factors. Akther et al. [20] and Wallace et al. [22] also found no significant correlation between sepsis and blood transfusion as risk factors for developing ROP. The study by Aydemir et al. [10] found sepsis and blood transfusion to have a significant correlation, but RDS and IVH were not found statistically significant.

In the present study, we also compared risk factors for Type 1 and Type 2 ROP and didn't find any statistically significant correlation between gestational age at birth, birth weight, and duration of oxygenation with the severity of ROP, which may be due to unequal distribution of the sample between the two groups (Type 1:Type 2, 2:24). There are various studies by Akther et al. [20], Wallace et al. [22], Dwivedi et al. [15], Gopal et al. [26], Charan et al. [11], and Maheshwari et al. [12] that found these risk factors to be strong predictors of the severity of ROP.

In the present study, other risk factors, like sepsis (p = 1.000), IVH (p = 1.000), blood transfusion (p = 1.000), and RDS (p = 1.000), had not been found statistically significant for the development of severe ROP (Type 1) due to the small sample size and unequal distribution of samples among Type 1:Type 2 ROP (2:23). In the study by Rao et al. [21], IVH was found as an independent risk factor for the severity of ROP. Another study by Kim et al. [23] didn’t find sepsis as a risk factor for the severity of ROP. Aydemir et al. [10] found sepsis as an independent risk factor for the severity of ROP. Wallace et al. [22] also found no statistical significance of blood transfusion and sepsis with the severity of ROP.

In the present study, the mean weight change (grams) was calculated for the first six weeks of postnatal life, and the mean weight change was found to be more significant in the fourth week of postnatal life. Some studies reported a relationship between poor postnatal weight gain and an increased risk of developing ROP. A study by Fortes Filho et al. [27] reported that low weight gain at six weeks was an essential and independent risk factor for developing ROP, which requires treatment.

The predictive power of the mean change in weight at four weeks for predicting the development of ROP was analyzed by plotting the ROC curve. The area under the curve was 0.77 (95% CI: 0.634-0.907). The discriminative power was fair.

The mean change in weight for Type 1 ROP and Type 2 ROP was compared for the first six weeks of postnatal life. We couldn't find any statistical correlation between the two groups, but we found that preterm babies with Type 1 ROP had lower weight gain than those with Type 2 ROP. The trend of mean change in weight between the fourth and sixth weeks is much lower than in the first three weeks in preterm babies with Type 1 ROP.

There were various studies showing postnatal weight gain as an independent predictor of the severity of ROP. A study by Wallace et al. [22] reported an independent association between the absolute postnatal weight gain (g/day) at six weeks of life and the severity of ROP. In another study by Kim et al. [23], poor postnatal weight gain (relative weight gain) in the first two weeks of life was found to be an important and independent risk factor for ROP requiring treatment.

In India, Rao et al. [21] were the first to highlight the relationship between postnatal weight gain and the severity of ROP. They found that the mean weight gain proportion was not significantly different at four, five, and six weeks, as in our study, but they used weight gain proportion. However, their study found that neonates with severe ROP had a poor weight gain proportion of 30% at six weeks. Another study by Wallace et al. [22] showed that preterm babies with Type 1 ROP had weight gain of less than 50% of their birth weight in the first six weeks of life. Similarly, in the present study, preterm babies with Type 1 ROP had a postnatal weight gain of less than 50%, which is in accordance with the previous study.

In this study, due to the unequal distribution of preterm babies with Type 1 ROP and Type 2 ROP, we were unable to do logistic regression analysis, so we can't comment on postnatal weight gain as an independent predictor for the severity of ROP. The strengths of this study include the prospective observational nature of the study, the methodological approach in investigating the relationship between weight gain and ROP, the use of rigorous ethical guidelines by ICROP, and screening and treatment performed by a single senior ophthalmologist with over a decade of experience in ROP screening, further validating the clinical assessment. The limitations of this study include a small sample size (n = 50), unequal distribution of preterm babies between Type 1 and Type 2 ROP, asymmetric distribution of preterm babies between inborn and out-born, and the association with other risk factors that were not analyzed. The majority of previous studies have analyzed various risk factors and weight gain with the occurrence of ROP, but none have documented the relationship with the occurrence of ROP. This is probably the first study analyzing the same from a tertiary care facility. In future studies, a larger sample size will be required to accurately comment on postnatal weight gain as an independent predictor of the severity of ROP. The limitations of the present study are the small sample size, potential confounders, and statistical limitations.

## Conclusions

The results of this study suggest that poor postnatal weight gain in the first four weeks of life plays a vital role in predicting the development of ROP. However, the mean weight change was lower in preterm babies with Type 1 ROP. Among other risk factors, not only the duration of oxygen delivery, but also the mode of oxygen delivery, has been highlighted as an important risk factor for the development of ROP.
